# A Description of a Curious Lusus Naturæ

**Published:** 1783

**Authors:** 


### III. *A description of a curious Lusus Naturæ. By Mr.* J. L. Green, *surgeon at Peckham in Surry.*

ON the 13th of February 1780, I was desired by a gentleman of the faculty to go and see a dead child of Mr. H. of East-lane, Bermondsey, to which he had been sent for the day before to examine and give his opinion, to which of the two sexes it belonged.

Upon examination there appeared two folds of integuments exactly similar to the labia pudendi, and lying between them a part resembling the penis, which, however, was attached all along from one extremity to the other, and could not be displaced by the fingers; so that it had the appearance of a female child with a penis attached to the os pubis, and hanging down between the labia. I could not at first discover the meatus urinarius, or any other orifice; but the nurse assured me that the child had made water, and upon preſſing very hard with my fingers upon the abdomen juſt above the pubis, urine was diſcharged from a very ſmall orifice immediately below what appeared to be the glans penis, covered by its prepuce, and immediately between what appeared like the labia pudendi. The glans penis could not be denuded. I thought I could feel the teſticles lying in the groin, but could not be certain; and the meatus urinarius terminating much in the like manner as in a female, puzzled me exceedingly; and I would not take upon me to decide poſitively, whether it was a boy or a girl, unleſs I diſſected it. However, it had been baptized as a female, and lived three days *.

Upon opening the abdomen I immediately diſcovered the ſpermatic cords paſſing through the rings of the external oblique muſcles. I traced theſe, and found a perfect teſticle, with

* "Graaf dit avoir vû une fille qui avoit le clitoris "dès ſa naiſſance ſi fort reſemblant au membre viril, "que la ſage femme et celles qui ſe trouverent à l'ac-"couchement de ſa mere, la crurent un garçon; et lui "firent donner au baptême un nom d'homme: mais "cette erreur fut decouverte après la mort de l'enfant, "en faiſant une exacte diſſection de ſon cadavre."

PALFIN.

its its regular ſet of veſſels on each ſide; the one on the right ſide lying in the groin; that on the left had deſcended into the labia, or what I ſhould now more properly call the divided ſcrotum. All the abdominal viſcera appeared in a ſound and natural ſtate, excepting the bladder, which was much thickened, contracted, and full of rugæ on its inſide. I was deſirous of having this extraordinary ſubject in my poſſeſſion, but this was impoſſible; I was therefore obliged to content myſelf with removing the bladder and external parts of generation.

Upon examining theſe I found that the urethra did not paſs through the penis, but ran nearly in a ſtrait line from the meatus urinarius into the bladder, and was about the ſame length as one would have expected to have found it in a female child of the ſame ſize. The proſtate gland was in its natural ſituation, as were the veſiculæ ſeminales; but I could not, from the minuteneſs of the parts, diſcover whether theſe opened into the urethra on each ſide of the verumontanum as uſual, or not. The raphée of the peritonæum was remarkably prominent.

Such a luſus naturæ as this would, I apprehend, in earlier times, have been deemed hermaphrodite, maphrodite, and indeed the external parts had very much the appearance of a combination of the fexes.

I was not permitted to examine the thoracic vifcera, but it was obferved, that the child had no difficulty of breathing, and therefore probably there was no mal-conformation in the cheft.

I believe fuch children would live longer than they ufually do, if proper care was taken; but it is not to be wondered at if parents and their attendants are not over anxious to preferve the life of fuch monftrous productions.

*Peckham,*
*July* 30, 1783.

